# Linguistic and Cultural Adaptation of a Computer-Based Counseling Program (CARE+ Spanish) to Support HIV Treatment Adherence and Risk Reduction for People Living With HIV/AIDS: A Randomized Controlled Trial

**DOI:** 10.2196/jmir.5830

**Published:** 2016-07-13

**Authors:** Ann E Kurth, Nok Chhun, Charles M Cleland, Michele Crespo-Fierro, José A Parés-Avila, John A Lizcano, Robert G Norman, Michele G Shedlin, Barbara E Johnston, Victoria L Sharp

**Affiliations:** ^1^ Yale University School of Nursing Orange, CT United States; ^2^ New York University Rory Meyers College of Nursing New York, NY United States; ^3^ University of Arizona College of Nursing Tucson, AZ United States; ^4^ New York University College of Dentistry New York, NY United States; ^5^ Lincoln Community Health Center Durham, NC United States; ^6^ St. Luke's-Roosevelt Hospital Center Center for Comprehensive Care New York, NY United States

**Keywords:** antiretroviral therapy adherence, computer-based counseling, cultural adaptation, HIV, linguistic adaptation, prevention with positives, Technology Acceptance Model, viral load

## Abstract

**Background:**

Human immunodeficiency virus (HIV) disease in the United States disproportionately affects minorities, including Latinos. Barriers including language are associated with lower antiretroviral therapy (ART) adherence seen among Latinos, yet ART and interventions for clinic visit adherence are rarely developed or delivered in Spanish.

**Objective:**

The aim was to adapt a computer-based counseling tool, demonstrated to reduce HIV-1 viral load and sexual risk transmission in a population of English-speaking adults, for use during routine clinical visits for an HIV-positive Spanish-speaking population (CARE+ Spanish); the Technology Acceptance Model (TAM) was the theoretical framework guiding program development.

**Methods:**

A longitudinal randomized controlled trial was conducted from June 4, 2010 to March 29, 2012. Participants were recruited from a comprehensive HIV treatment center comprising three clinics in New York City. Eligibility criteria were (1) adults (age ≥18 years), (2) Latino birth or ancestry, (3) speaks Spanish (mono- or multilingual), and (4) on antiretrovirals. Linear and generalized mixed linear effects models were used to analyze primary outcomes, which included ART adherence, sexual transmission risk behaviors, and HIV-1 viral loads. Exit interviews were offered to purposively selected intervention participants to explore cultural acceptability of the tool among participants, and focus groups explored the acceptability and system efficiency issues among clinic providers, using the TAM framework.

**Results:**

A total of 494 Spanish-speaking HIV clinic attendees were enrolled and randomly assigned to the intervention (arm A: n=253) or risk assessment-only control (arm B, n=241) group and followed up at 3-month intervals for one year. Gender distribution was 296 (68.4%) male, 110 (25.4%) female, and 10 (2.3%) transgender. By study end, 433 of 494 (87.7%) participants were retained. Although intervention participants had reduced viral loads, increased ART adherence and decreased sexual transmission risk behaviors over time, these findings were not statistically significant. We also conducted 61 qualitative exit interviews with participants and two focus groups with a total of 16 providers.

**Conclusions:**

A computer-based counseling tool grounded in the TAM theoretical model and delivered in Spanish was acceptable and feasible to implement in a high-volume HIV clinic setting. It was able to provide evidence-based, linguistically appropriate ART adherence support without requiring additional staff time, bilingual status, or translation services. We found that language preferences and cultural acceptability of a computer-based counseling tool exist on a continuum in our urban Spanish-speaking population. Theoretical frameworks of technology’s usefulness for behavioral modification need further exploration in other languages and cultures.

**Trial Registration:**

ClinicalTrials.gov NCT01013935; https://clinicaltrials.gov/ct2/show/NCT01013935 (Archived by WebCite at http://www.webcitation.org/6ikaD3MT7)

## Introduction

Human immunodeficiency virus (HIV) disease in the United States disproportionately affects minorities, including Latinos [1]. Barriers such as language are associated with lower antiretroviral therapy (ART) adherence among Latinos, yet ART and interventions for clinic visit adherence are rarely developed or delivered in Spanish. Although treatment of HIV has advanced tremendously with the development of ARTs, these medication regimens require lifelong adherence to achieve therapeutic goals [2-5].

The computer-based counseling tool known as the Computer Assessment & Rx Education for HIV-positives (CARE+) is an evidence-based intervention for people living with HIV and acquired immune deficiency syndrome (AIDS) (PLHA) [6,7]. The purpose of the program is to support users in achieving medication adherence and reduce their risk of secondary HIV infections (also known as “positive prevention”). This program, when evaluated in a university-affiliated public HIV clinic and a community-based AIDS service organization in an English-speaking population in Seattle, was found to be effective in reducing HIV-1 viral load and sexual transmission risk behaviors [6]. Technology tools such as CARE+ present significant opportunities to bridge the gap in health promotion delivery, especially if linguistically and culturally adapted for often-neglected groups such as Latinos. In this paper, we use the term Latino; however, in the literature, Latino and Hispanic are used interchangeably, reflecting a lack of consensus as well as the political and demographic implications of both terms [8].

Latinos make up approximately 17% of the US population, but according to the Centers for Disease Control and Prevention, account for 23% of all new HIV infections reported in 2013 in the United States [9]. Furthermore, Latino men account for 85% of all new infections among Latinos in the United States and 81% acquired HIV infection through sexual contact with another male [9]. In contrast, in 2013, Latino women accounted for 15% of all new infections among Latinos in the United States [9]. It is estimated that one in 36 Latino men and one in 106 Latino women will be diagnosed with HIV at some point in their lives [1].

**Figure 1:**
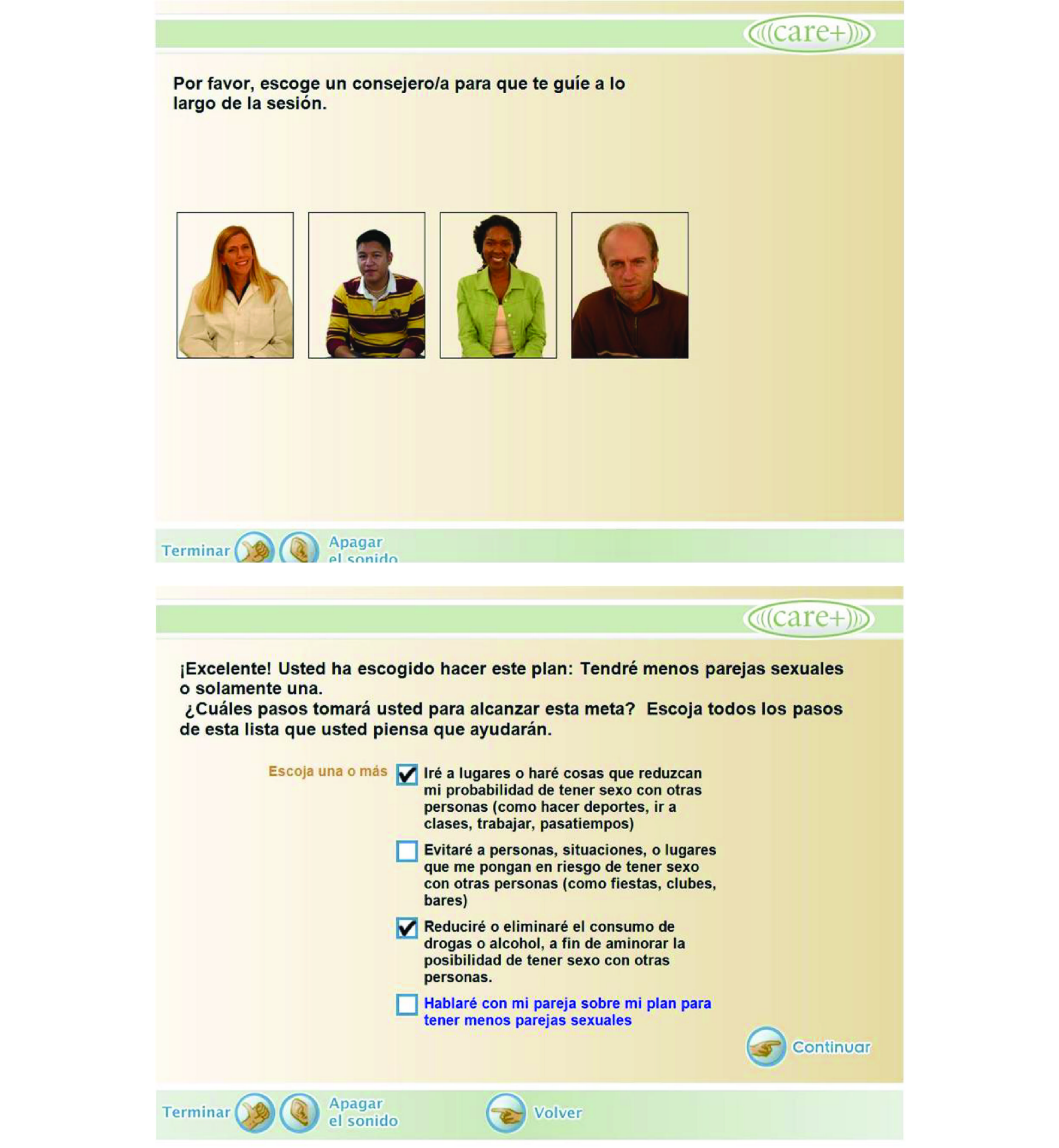
Screenshots of CARE+ Spanish.

In this manuscript, we describe the adaptation of the CARE+ tool for a Spanish-speaking population (CARE+ Spanish; Figure 1). The Technology Acceptance Model (TAM) [10] was the conceptual framework that guided the Spanish adaptation of the computer-based counseling program. New information and communication technologies (ICT) must be culturally acceptable if they are to be effective in daily clinical practice, rather than just in the context of a controlled trial. Acceptability is defined as the “degree to which a[n]...intervention or any one of the attributes of the...intervention is perceived by the patient/consumer to be consonant with well-being” [11]. Acceptability centers on perception of an innovation (ie, ICT tool) as clusters of perceived attributes. These can be categorized as follows, using ART as an example: (1) perceived inherent attributes (eg, ART is effective or requires scheduling); (2) perceived associational attributes (eg, ART demands periodic clinic visits; is encouraged by medical personnel, but perhaps not by others); and (3) perceived effects (eg, lifestyle and other changes). Each perceived attribute has cultural meaning, and the individual continually weighs the positive and negative aspects of the attributes throughout treatment. The resulting balancing act influences the acceptability of the intervention. Cultural and linguistic factors determine the perception of relevancy of these attributes, as well as other factors, such as age, life cycle stage, health status, motivation to be healthier, and perceptions of the source of the information and intervention [11,12].

The study aims were to (1) conduct usability testing of CARE+ Spanish; (2) establish a real-world utility of CARE+ Spanish by conducting a 12-month longitudinal randomized controlled trial (RCT) to evaluate the impact of the CARE+ Spanish intervention on outcomes, which included ART adherence, sexual transmission risk behaviors, and HIV-1 viral loads; and (3) to assess technology uptake factors, explore the cultural acceptability of the tool, and perceived technology barriers/facilitators among participants and health care providers using the TAM framework.

## Methods

The CARE+ program incorporates motivational interviewing and principles of chronic HIV disease self-management to enhance health-promoting behavior. Formative research to test the usability and acceptability of the CARE+ computer-based counseling tool has been previously described [7], and the tool was shown in an English-speaking population to improve ART adherence, viral suppression, and reduced secondary sexual transmission risk behavior [6]. Given that computer-based counseling proved promising in an English-speaking population in Seattle, translation into practice and applicability to other populations that could benefit, such as Spanish-speaking Latinos or Spanish-dominant bilingual Latinos living with HIV, guided the adaptation of the CARE+ tool for a Spanish-speaking population.

The recommended process for adaptation and translation from English to a different language version is the forward-back translation method in order to ensure cultural and linguistic equivalence [13]. Given budgetary constraints, and the large amount of software content, an expert panel review was utilized in place of back translation of the CARE+ tool. The expert panel consisted of clinical experts who were bilingual and bicultural with ancestry from major Latino subgroups (ie, Mexican and Puerto Rican). For the forward translation, a translator with a master’s degree experienced with HIV health-related materials translated the CARE+ content into Spanish. Because the professional translator’s work had been used with predominantly Mexican-American populations, a member of our study team (JAP), who is of Puerto Rican origin and has experience in AIDS research with Mexican and Puerto Rican populations, took on the role of assuring quality of translation and applicability across Latino subgroups. Additionally, there was a secondary reviewer, a health educator and curriculum writer with a master of public health, who has done substantial translation work for HIV interventions in California, Florida, Puerto Rico, and the Northeastern United States. She performed final reviews of the translated software content. Spanish-speaking actors of Mexican and Colombian heritage recorded the narration, and text changes were also made if the two voice actors reading the script made recommendations. Then, a panel of bilingual HIV health care providers (two physicians, a nurse practitioner, a nurse, and a health educator) reviewed the translation of the content independently to confirm the appropriateness of the terminology and the minimization of idiomatic regional expressions unique to their culture. After discussion to reach consensus, words identified as idiomatic were deleted and substituted with words that were not specific to only one Latino subgroup. Furthermore, an additional local expert advisory panel (composed of one Spanish-speaking person living with HIV, and HIV providers from medicine, nursing, and social work) was convened in New York City to review the CARE+ Spanish tool content and shorten it for use in a real-world high-volume HIV clinic setting. Once a test-ready version of CARE+ Spanish was available, the program was tested with HIV-positive Spanish-speaking individuals to explore acceptability and usability of the intervention. Feedback from the usability testing was incorporated into the final version of the CARE+ Spanish program used for the RCT. In addition, we conducted exit interviews to explore cultural acceptability of the tool among purposively selected intervention participants, and focus groups among clinic providers to explore program acceptability and system efficiency issues.

### Participants

#### Usability Testing

Participants for the usability portion of the study were recruited and verbally consented from St Vincent’s Catholic Medical Center HIV clinic in New York City the last week of March 2010 (IRB #09-096). Because we were interested in the participants’ opinions about CARE+ Spanish, and not its efficacy or effectiveness, participants were informed that they could make up answers and skip questions to avoid disclosing personal health information. Using the “think aloud” method [14,15], participants were observed as they completed the CARE+ Spanish program. The observation was timed and careful notes were taken, paying attention to any difficulties navigating the program or understanding the content. While working with the program, the participant was encouraged to “think aloud” and share thoughts in their own words as they worked through the various tasks. A series of structured questions and verbal prompts were also used to elicit participants’ reactions to the program. Participants were given a US $20 MetroCard for their time and to reimburse their transportation costs. As a result of St Vincent’s closing in April 2010, the study was moved to St Luke’s-Roosevelt Hospital for the initiation of the RCT and remainder of the project.

#### Randomized Controlled Trial

Study participants for the RCT were recruited in the waiting areas from three urban HIV clinic sites of St Luke’s-Roosevelt Hospital in New York City from June 4, 2010 to January 3, 2011. Eligibility criteria were (1) adults (age ≥18 years), (2) Latino birth or ancestry, (3) speaks Spanish (mono- or multilingual), and (4) on antiretrovirals at any of the three clinic study sites.

Written informed consent was obtained from all RCT participants. All study procedures were approved by St Luke’s-Roosevelt Hospital, Center for Health Sciences, Institutional Review Board (#10-068) and New York University School of Medicine’s Institutional Review Board (#09-0740). This RCT is reported according to the CONSORT checklist [16] and the CONSORT-EHEALTH extension [17] (Multimedia Appendix 1). The RCT participants received a US $20 MetroCard for their time and to reimburse their transportation costs at the end of each study visit (five sessions total and an additional US $20 MetroCard if they participated in the exit interview).

The RCT participants were enrolled by research assistants, some of whom were part of the HIV-positive peer program at St Luke’s-Roosevelt Hospital. Participants of the peer program were Spanish-speaking PLHA, who were also receiving care at the St Luke’s Roosevelt Hospital HIV clinics. Peers in the program were selected as study staff based on their language skills and experience working with other Spanish-speaking PLHA in the HIV clinics. All research assistants completed human subjects’ protection certification, which included Health Insurance Portability and Accountability Act (HIPAA), and received training on the study protocol and procedures, and the use of the CARE+ Spanish program.

#### Focus Groups

The health care providers who participated in the focus groups were engaged in the care of participants (eg, prescribed antiretrovirals and/or supported ART adherence) and were recruited from the same three urban HIV clinic sites of St Luke’s-Roosevelt Hospital in New York City as the RCT participants. The two focus groups took place on February 21, 2012 and February 28, 2012. Participants in the health care focus groups were a variety of psychological and medical professionals who had provided care to PLHA for a wide range of years.

### Intervention

The CARE+ Spanish computer-based counseling program was delivered on touchscreen computers with content based on the following theoretical frameworks: information-motivation-behavior [18], social cognitive role modeling [19], and motivational interviewing [20]; and it was evaluated in a prospective longitudinal two-arm RCT design. Participants were automatically randomized by the software to the control or intervention arms following an anonymous study log in by the user. All participants were guided through the program with audio narration of all content. The intervention session lasted approximately 45 to 60 minutes; the control session lasted approximately 20 to 30 minutes. The control group received only the computer-based audio-narrated risk assessment, which included questions about sexual risk behaviors, substance use, mental health, social support, partner status and disclosure, ART regimen and adherence in last 7 and 30 days, and side effects. In addition to the computer-based audio-narrated risk assessment, the intervention group received tailored feedback through the skill-building videos, health plan, and printout at the end of the session, within the CARE+ Spanish software program. The skill-building videos automatically launched; afterwards, participants could choose to watch additional videos. Video topics included demonstration of healthy behaviors, such as condom use and medication adherence, and discussions about HIV and provider relationships. In the final step, users developed a risk reduction plan related to either ART adherence or safer sex practices to prevent secondary HIV transmission. After the risk reduction plan, participants could opt to watch more videos. At the conclusion of the session, participants received a printout of their tailored feedback and health promotion plan that they could share with their health care provider.

Both study arms also received standard clinical care per Department of Health and Human Services HIV Guidelines [2]. In addition, the computer-based tool identified participants who were experiencing severe depression as measured by the Patient Health Questionnaire (PHQ-9; score ≥20) [21], intimate partner violence, or suicidal ideation. Per study protocol, case managers were then notified for appropriate follow-up and referral. Each group underwent five sessions total at 3-month intervals (at 0, 3, 6, 9, and 12 months). At the 12-month session, the control group was switched over to the intervention condition. Study sessions were scheduled to coincide with clinic visits whenever possible.

### Exit Interviews

The purpose of the qualitative exit interview (Multimedia Appendix 2) was to explore the cultural acceptability of the tool and assess perceived technology barriers and facilitators among participants. Exit interviews were offered to purposively selected intervention participants using systematic sampling. At the 12-month session, every third eligible candidate was offered a face-to-face exit interview. We purposively sampled females and males, older (age ≥35 years) and younger (age ≤35 years), and US- or foreign-born participants. Exit interviews were performed using a semistructured interview guide by research assistants in either English or Spanish as requested by the participant. The interview guide was developed to explore the main concepts of TAM and other cultural factors known to impact the Latino community, such as stigma related to HIV, language, health insurance coverage, and immigration status. Notes were taken by research assistants in English and/or Spanish, including verbatim quotes to capture illustrative comments from respondents.

### Health Care Provider Focus Groups

Health care providers who participated in the focus groups gave written informed consent and received US $50 for their time and travel. Two focus groups were conducted; the sessions lasted approximately 2 hours. The focus groups were conducted using a semistructured interview guide (Multimedia Appendix 3), which outlined topics to be discussed along with suggested probes. Topics included challenges that providers faced in delivering care to HIV-positive patients, adherence-related issues, and usefulness of the CARE+ Spanish computer-based counseling tool. To improve attendance of the providers, the focus groups were conducted before required staff meetings. Prior to the start of the focus group, the providers were given an opportunity to view the counseling program (tablet and headphones), as well as a sample session printout. Due to scheduling issues, different teams of study staff conducted the two focus groups.

### Outcome Measures

The primary outcome was HIV-1 viral load collected from medical chart reviews. This biomarker, along with adherence to medications (measured by 30-day visual analog scale [VAS]) and sexual transmission risk behaviors (defined as lack of condom use with either a main or other partner) identified through the CARE+ Spanish program, were the outcome measures for the assessment of intervention effectiveness. These outcome measures were collected at 0, 3, 6, 9, and 12 months. Although the outcome measures were not collected from every participant at each time interval, a minimum of three data points were collected from all participants.

### Sample Size Determination

For the usability testing, sample sizes greater than five participants have been shown to have sufficient power to detect the majority of usability problems [22]. For the RCT, sample size was calculated based on the target intervention effect on the proportion of participants who are ART adherent, HIV viral load at log10 scale, and occurrence of unprotected sex with HIV-negative/unknown partner(s). All calculations control type I error rate at 0.05. Considering a decrease of 0.5 log10 HIV viral load as a meaningful reduction, with 200 retained participants in each group in a time point-specific post hoc test, there was an expected >97% power to detect this difference with a standard deviation of 1.25 (effect size=0.4).

### Statistical Analysis

Fisher exact and Wilcoxon rank sum tests assessed differences between intervention and control groups in population study characteristics at the baseline assessment. Linear and generalized mixed linear effect models were used to longitudinally assess differences between the intervention and control groups at all available time points for sexual transmission risk behavior, medication adherence (30-day VAS), and viral load variables. These models accommodate missing data (equal numbers of measurements and time intervals between measurements were not required) and, therefore, do not require deletion of participants with incomplete data. A *P* ≤.05 was used as the cutoff for significance. Primary outcomes included ART adherence, sexual transmission risk, and HIV-1 viral loads. Baseline analyses were performed using SAS version 9.3 (SAS Institute Inc, Cary, NC, USA) and, for the longitudinal analysis, the lme4 package [23] of the R statistical computing environment [24]. Given that the control group received the intervention at 12 months, data analysis was limited to four time periods (0, 3, 6, and 9 months). Effect sizes for undetectable viral load and sexual transmission risk were presented in the odds ratio metric. For viral load and 30-day VAS, Cohen’s *f*^2^ was calculated to convey effect sizes for group differences in change over time [25].

### Interview Analysis

Data from the exit interviews were transcribed onto spreadsheets by two researchers from the study team (MGS and MCF), while the focus groups were recorded by a stenographer, with transcripts provided from the two sessions. Data were analyzed using content analysis within a framework of technology transfer [10] to identify factors affecting acceptability, utilization, and impact. The exit interview spreadsheets and the focus group transcripts were analyzed by MS and MCF; emergent themes and issues were categorized by each. Saturation of themes was determined after 61 participant exit interviews and the two provider focus groups. Inconsistencies in the themes were discussed between MS and MCF until consensus was reached; selected quotations were agreed on as salient examples of themes.

## Results

### Usability Testing

Software usability testing was conducted with eight Spanish-speaking PLHA (6 male, 2 female). Five of six males identified their language preference as bilingual and one as English-dominant; one female identified as bilingual and the other as English-dominant. All usability participants reported the program was easy to use and navigate; questions were clear, specific, and understandable. All participants who identified as bilingual (6/8) reported that the Spanish used was basic and easy to understand. The two English-dominant participants reported there were some “big (high-register) words,” but they were able to navigate the program and follow instructions without any major problems. All participants agreed that the counseling tool supported privacy and confidentiality, especially for people who are more quiet and reserved about their HIV status. They agreed when using this counseling tool, one can be more open and honest about responses because of the feeling of not being judged. Seven of eight reported that they would prefer to use this tool rather than counseling with a person. Overall, the counseling tool met with everyone’s expectations; on average, it was rated a nine out of 10. It was described as user friendly and self-explanatory.

### Randomized Controlled Trial

**Figure 2:**
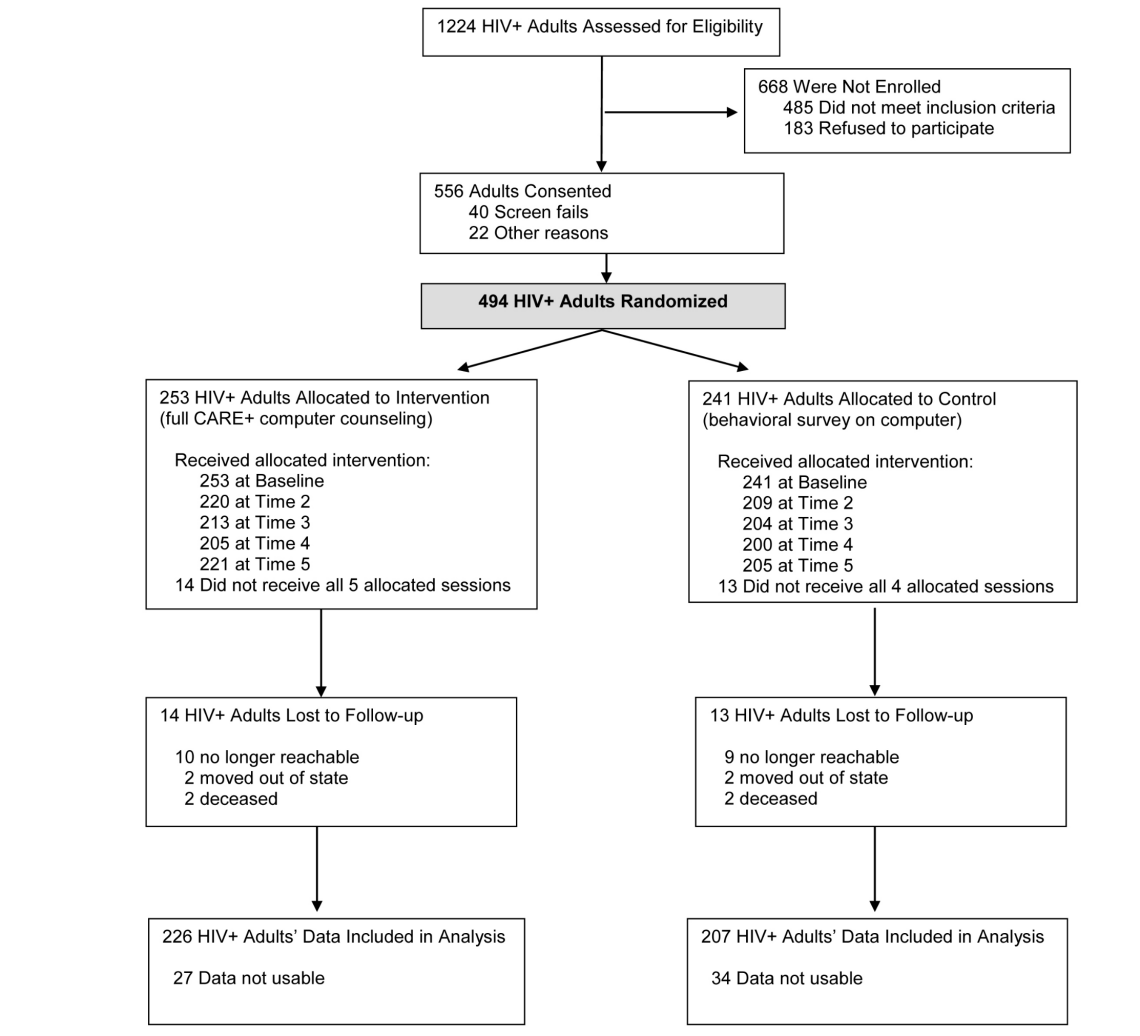
Participant flowchart of the CARE+ Spanish computer-based counseling intervention trial, five sessions over 12 months.

We approached 1224 individuals at three study sites; 556 consented (45.42% acceptance), 494 were randomized and completed baseline assessments, and 86.2% (426/494) were retained for the 12-month study duration (Figure 2).

Table 1 illustrates participant characteristics at baseline by study arm. There were no significant differences between the treatment and control groups at baseline except that the CARE+ intervention group had a higher proportion of transgendered individuals (4.0%, 9/225 vs 0.50%, 1/206; *P*=.05) and were younger than those in the control group (mean 46.8, SD 9.7 vs mean 48.9, SD 9.1; *P*=.02, respectively).

**Figure 3:**
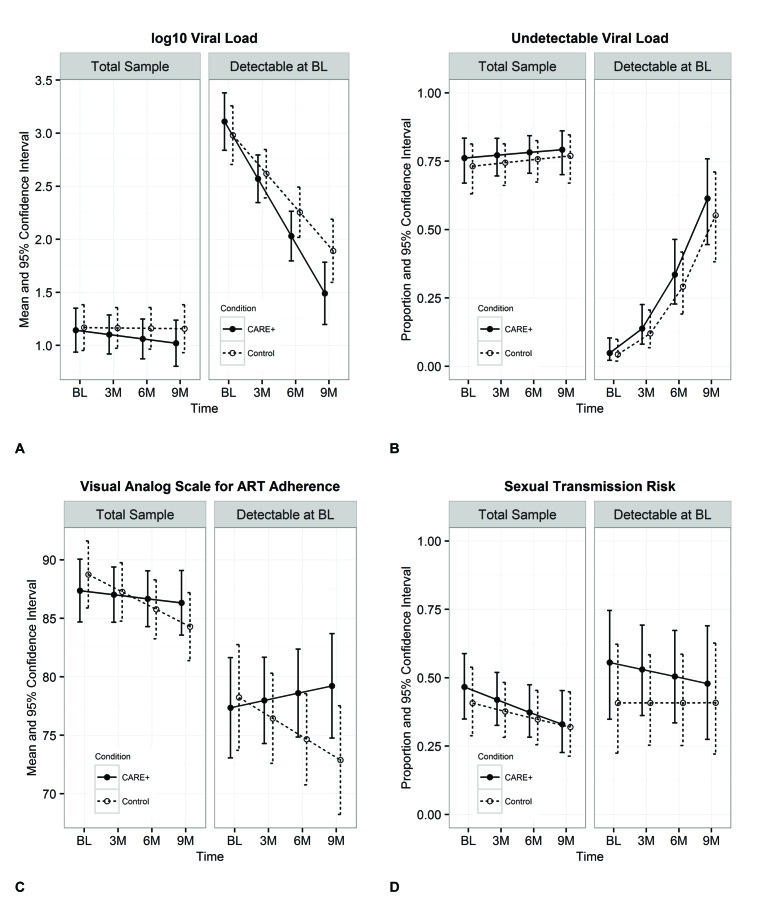
Adjusted mean values or proportions by time and treatment condition of the total sample and those with detectable viral loads at baseline (BL) for (a) log10 viral load, (b) undetectable viral load, (c) visual analog scale for ART adherence, and (d) sexual transmission risk. Whiskers represent 95% confidence intervals.

Figure 3 shows the mean and 95% confidence intervals for outcome means or proportions by time point and treatment condition. Although intervention participants had reduced viral loads, increased ART adherence, and decreased sexual transmission risk behaviors over time, patterns of change in the intervention group were not more favorable than in the control group.

**Figure 4:**
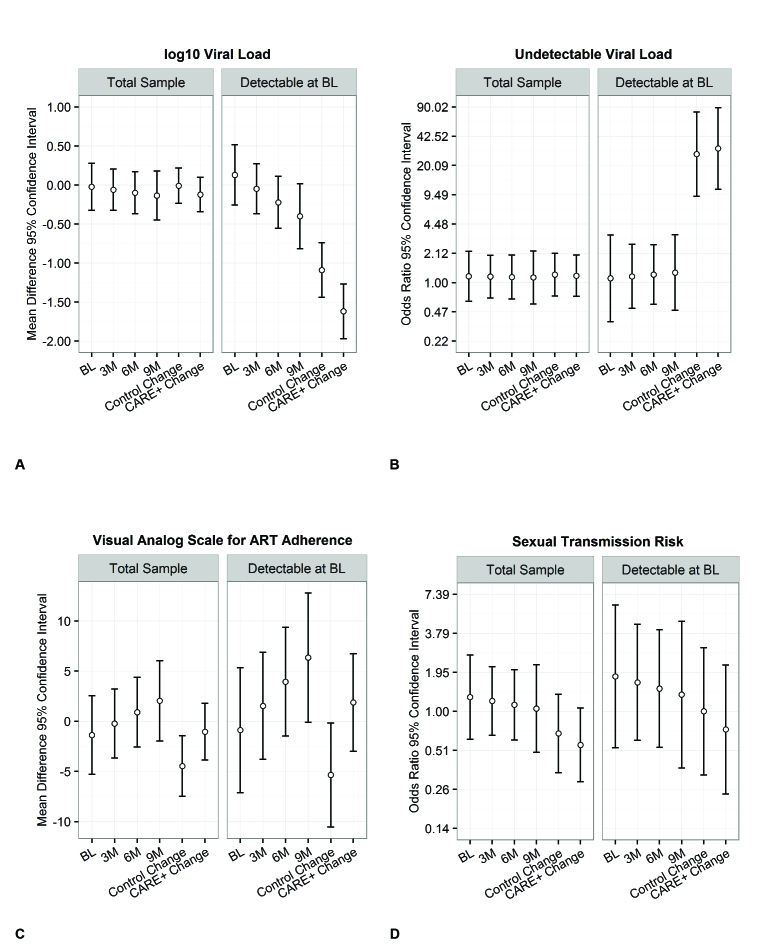
Mean differences or odds ratios contrasting CARE+ and control conditions at each follow-up time point, and baseline (BL) and final time points within each condition, for (a) log10 viral load, (b) undetectable viral load, (c) visual analog scale for ART adherence, and (d) sexual transmission risk. Whiskers represent 95% confidence intervals.

Figure 4 summarizes main outcomes of interest at each follow-up time point. Figure 4 A illustrates 95% confidence intervals for log10 viral load mean differences and change at each different time point as well as overall change in the control versus the CARE+ intervention group. Although there was a decrease in viral loads among participants with a detectable load at baseline (greater in the intervention vs control group), this difference in change was not statistically significant. In addition, among those with detectable viral loads at baseline, the CARE+ intervention group had higher odds of being undetectable at the 9-month follow-up when compared to controls (Figure 4 B), but this difference was not statistically significant. Figure 4 C shows 95% confidence intervals for VAS differences at each follow-up time point; although ART adherence was higher in the CARE+ intervention group vs control in the total sample overall and among participants with detectable viral loads at baseline, no differences in change or at any follow-up point were statistically significant. Finally, although sexual transmission risk behaviors decreased over time, when intervention and control groups were compared, no differences in change or at any follow-up point were statistically significant.

Differences between the CARE+ intervention group and the control group in change on viral load were small both for the total sample (*f*^2^=0.0003) and for those with detectable viral load at baseline (*f*^2^=0.006). Similarly, group differences in change on VAS were small for both the total sample (*f*^2^=0.002) and for those with detectable viral load at baseline (*f*^2^=0.005). Figures 4 B and 4D show effect sizes for the sexual transmission risk and undetectable viral load outcomes in the odds ratio metric. Differences in undetectable viral load between the CARE+ intervention group and the control group were consistently small at baseline and across follow-ups. Differences in sexual transmission risk were small at baseline and became even smaller with each follow-up.

**Table 1:** Demographic characteristics of CARE+ Spanish intervention and control groups (N=433).

Variable		CARE+ Spanish (n=226)	Control (n=207)	*P* ^a^
**Sex, n (%)**				
	Male	153 (68.0)	143 (69.4)	.05
	Female	56 (24.9)	54 (26.2)	
	Transgender	9 (4.0)	1 (0.5)	
	Unknown	7 (3.1)	8 (3.8)	
**Age (years), mean (SD)**		46.8 (9.7)	48.9 (9.1)	.02
**Ethnicity, n (%)**				
	Latino	218 (96.9)	191 (92.7)	.20
**Race, n (%)**				
	American Indian or Alaska Native	12 (5.3)	13 (6.4)	.33
	Asian	2 (0.8)	0 (0.0)	
	Black or African American	13 (5.8)	19 (9.2)	
	Native Hawaiian or other Pacific Islander	2 (0.8)	3 (1.5)	
	White	38 (16.8)	42 (20.4)	
	Other race	151 (66.8)	118 (57.3)	
	Multiple race	3 (1.3)	5 (2.4)	
	Unknown	5 (2.2)	7 (3.4)	
**Education, n (%)**				
	No high school diploma/GED	79 (35.0)	72 (34.8)	.13
	High school diploma/GED only	65 (28.8)	75 (36.2)	
	More than high school	75 (33.2)	56 (27.1)	
	Unknown	7 (3.1)	4 (1.9)	
**Substance use behavior, n (%)**				
	Ever injecting drug use	12 (5.3)	10 (4.8)	.55
	Alcohol abuse	44 (19.6)	41 (19.8)	>.99
	Methamphetamine use	12 (5.5)	12 (6.1)	.84
	Crack/cocaine use	24 (11.2)	25 (12.6)	.76
Intimate partner violence, n (%)		16 (7.2)	—	—
**Sexual behavior, n (%)**				
	Any sex past 3 months	139 (63.8)	124 (62.3)	.76
	Risky sex^b^	35 (25.9)	26 (21.7)	.46
	Condom use with problems	47 (33.8)	36 (29.0)	.43
	Any sex without condoms or with condom problems	69 (50.7)	56 (46.3)	.53
	Discordant sex with main partner	8 (3.7)	7 (3.5)	>.99
	Discordant sex with other partner	11 (5.1)	6 (3.1)	.46
**ART Adherence**				
	Adherence VAS, mean (SD)	87.0 (22.4)	89.6 (19.5)	.20
	VAS Scale ≥95%, n (%)	135 (59.7)	122 (58.9)	.92	
	Missed doses, mean (SD)	1.5 (8.0)	1.6 (8.2)	.86
	1 or more missed doses past 7 days, n (%)	74 (37.2)	64 (35.2)	.75
	2 or more missed doses past 7 days, n (%)	41 (20.6)	36 (19.8)	.90
	> VAS 95% + no missed doses, n (%)	93 (44.1)	89 (44.9)	.92
Log10 HIV-1 viral load, mean (SD)		1.2 (1.8)	1.2 (1.6)	.93
Detectable viral load, n (%)		78 (34.5)	76 (36.7)	.69
Ever told resistant virus, n (%)		30 (13.8)	32 (15.9)	.67
Years since HIV diagnosis, mean (SD)		12.6 (6.9)	13.4 (7.3)	.23
Depression (PHQ-9), n (%)		35 (15.6)	39 (18.8)	.38

^a^ Comparisons by Fisher exact test or Wilcoxon rank sum test; All categorical tests performed without the unknown category.

^b^ Did not use a condom with either main partner or other partner.

### Exit Interviews

The open-ended exit interview identified a range of HIV-related concerns, lack of confidence in providers, multiple sources for HIV-related information, and experiences using the CARE+ Spanish computer-based counseling program.

Characteristics of exit interview participants (N=61) are summarized. In all, 37 (61%) men, 21 (34%) women, and 3 (5%) transgendered persons participated in the interviews. Participant ages ranged from 21 to 69 years, with a mean age of 48.0 (SD 12.0) years. The participants included those born in the United States and Puerto Rico, and immigrants from Colombia, Costa Rica, the Dominican Republic, Guatemala, Honduras, Mexico, Nicaragua, and Panama, with a range of 4 to 50 years residing in the United States.

#### Concerns Related to Human Immunodeficiency Virus

In total, 95% (58/61) had disclosed their HIV status to someone. The main issues affecting the interviewed participants included insurance/benefits-related issues, side effects from medications (both antiretrovirals and other medications), being able to work, immigration documentation, and housing issues. The majority of participants did not feel that their issues related to medications or the virus were any different because they were Latino. However, some did feel there was a difference if the issue(s) related to documentation (ie, “having papers”). One participant noted, “illegal status, it leaves us without options.” Another participant shared additional comments regarding obstacles faced by their undocumented status: “Of course, because we are immigrants and we don’t have legal papers to get around or anything.” Some noted that English-speaking/non-Latinos have more information and receive different care. Other participants discussed the problems Latinos face that keep them from focusing on their illness, fears of engaging in care, not trusting to reveal their diagnosis, parents not teaching their children about safer sex, and fears relating to stigma and discrimination: “Spanish people appear stronger around other(s) and don’t share their weakness” and “Latinos have machista behavior in not taking care of themselves...” Language barriers were a concern in accessing information, understanding, and being understood by providers and navigating the health care system.

#### Multiple Sources of Information Related to Human Immunodeficiency Virus

Participants reported a range of resources for obtaining HIV-related and general health information. Overwhelmingly, doctors and clinic staff were reported to be reliable sources of information because they were said to have studied and practiced HIV care for some time: “I put my health in her hands, because she knows what is good and what isn’t for me.” (Note that participants may be referring to their prescribing providers as “doctors” whether they are medical doctors, nurse practitioners, or physician assistants.)

Some participants purposefully seek out other sources for HIV and general health information. The Internet, because “broader information (is) available,” and the Centers for Disease Control and Prevention “made the first concrete studies on managing care” were more trusted sources for some clients. Others cited support groups and peers at AIDS service organizations and clinics as expert voices for comparison of experiences. The media (radio, television, and print) were identified as potential sources of information for the general public. Lastly, families, friends, and spiritual leaders were seen as another source of information for some.

A majority of participants reported that they did receive messages from their providers regarding the importance of taking their medications regularly, having a sexual life with HIV/AIDS, and measures to prevent the transmission of HIV to others. Some participants reported not receiving medication information from their providers: “They have never told me anything.” Others knew that medications lessen viral loads, increase CD_4_ counts_,_ avoid creating resistance, reduce infections, and maintain overall health. One participant added, “It is a marriage with the medications.” Some participants were motivated to have these discussions about taking the medications because they reported needing to be there for their children and to maintain their quality of life.

For some participants, any discussion of sexual behavior was one they were not comfortable having with their providers. A few reported giving up on relationships and sex after receiving their HIV diagnosis. Overall, there was a clear awareness that condoms should be used to protect themselves and their partners from HIV and other sexually transmitted infections. Communication was discussed as another way to enhance protection. One of the participants stated that he did not use condoms with his wife who was also HIV-positive. Another discussed using withdrawal when his viral load is undetectable. A few participants stated that their providers did not discuss this topic with them. The participants’ answers were similar when discussing modes of reducing transmission (ie, condom use and open discussions with partners). They added abstinence and masturbation as ways to practice safer sex. Medicines were also seen as helping reduce transmission to infants.

For the most part, the majority of participants were comfortable discussing medications and sex with their providers. Some noted that they initiated discussions on medications and sex, whereas for others it was their providers. Some stated that they had “confidence” in their providers, whereas others felt it was necessary for their own well-being to take the initiative: “After receiving treatment with the same doctor for so long, this person becomes part of your family.” A salient finding was that some participants did not discuss medications with their providers because they felt they had other resources for information. Regarding sex, some reported preferring to speak with a provider of the same sex or same sexual orientation. A few did not have any discussions regarding medications or sex with their providers: “They don’t have the time.” Another participant summed up her sexual situation and the need to discuss it as follows: “There’s not much to say, my status was so traumatic that I don’t believe in love.”

#### CARE+ Spanish Computer-Based Counseling Program

The majority of participants reported a positive experience with using the tablet computer. They thought it was “interesting,” “easier to talk to than a person,” “it was like having another doctor,” “better than having a piece of paper,” “the computer doesn’t judge,” and “I educated myself and came out of such ignorance.” On the other hand, some had some issues with the questions being repetitive and the structure of the sentences. Participants reported learning more about HIV, their medications, and things they would not think of asking the provider.

“Likes” of the computer included the ease of use, the touchscreen, the videos, the confidentiality (through the headphones), and the information provided about medications, safer sex, the narrators, and avatars. “Dislikes” of the computer were the long sessions, computer problems such as taking a long time to reboot, the videos, the headphones, not knowing how to silence the program, and not having an option for English. Some participants were not comfortable with the directness of the language and some of the topics (drugs, sexual abuse). One participant noted, “Some questions were very strong, too direct, and a bit long.” The most salient objection, however, was the perceived redundancy of the content.

All the participants reported a sense of privacy and confidentiality while using the computer. Some were concerned about this at the beginning of the study and this was noted to be one of the reasons for some anxiety when first using the computer. Other reasons for anxiety at the start of the study were being unfamiliar with computers and unsure of Spanish language proficiency. Participants spoke of becoming familiar with the format of the program and having the peers to help them get used to it. The availability of the peers was viewed as an overwhelmingly positive aspect of the program.

Although the majority of those interviewed preferred the program in Spanish, some would have preferred to have an English version available and some thought having both languages would work best for them. (This is important to note for any replication in an urban environment where the population is likely to include acculturated and bilingual individuals.) Nearly all participants stated that they would use the program again to pass the time while waiting in the clinic, and when new information could be provided. Some would use it at every visit, whereas most opted for a few times a year. A few participants were clear in not wanting to use the computer program again; one person disliked the voice of the narrator and the other was uncomfortable with the computer. One participant stated that the program might be good for new clients at the clinic or newly diagnosed people.

Overall, the CARE+ Spanish program was viewed positively. Most participants used it to improve their health, learn their medications (eg “Showed me how much I know, tested me on what I knew, allowed me to be honest with myself”), and change some behavior (eg “I loved it! I learned so much and because of this I slowed down my sex life and am more careful”). Although some participants saw the program as an important part of their care by asking questions of their providers, others did not, seeing it as not relevant to their lives (eg “the long explanations of topics that were not relevant to me”). The spectrum of responses received in the exit interview reflects the diversity of the study population, Latinos living in New York City.

### Health Care Provider Focus Groups

The first focus group was conducted on February 21, 2012 with seven participants. Participants included three psychologists, three psychiatric nurse practitioners, and one psychiatrist. Years in health care ranged from 4 to 23 years and years in HIV care ranged from 1.5 to 20 years. There were three bilingual providers present. Providers represented all three study sites; time at their respective sites ranged from 1.5 to 15 years.

The second focus group was conducted on February 28, 2012 with nine participants, but only eight participants provided their demographic information. Participants included five medical doctors and three nurse practitioners. Years in health care ranged from 14 to 29 years and years in HIV care ranged from 11 to 14 years. There were three bilingual providers present. Providers represented all three study sites and time at their respective sites ranged from 5 months to 16 years.

Providers expressed frustration in continuing to confront the same adherence obstacles over time (eg, lack of consistent safer sex practices). However, this was a general finding and not specific to Latino patients. One provider reported that not using condoms makes the patient feel “normal” (eg, “I don’t feel sick with HIV when I don’t use a condom”). Other adherence obstacles that are in agreement with findings from the RCT exit interviews include substance abuse, lack of documentation, stigma, and trauma. According to one provider, trauma is a “huge, huge factor” because it affects people, their belief in their right to protect themselves, and their expectations that intimate encounters are at least safe for both parties. Overall, providers were in agreement that “like a hydra head,” when one issue gets addressed, another comes up.

Regarding the usefulness of the CARE+ Spanish tool, providers agreed that multiple approaches are important (eg “more education is always good”) and that patients may feel more empowered to have a resource (eg “an education tool” and “a health enhancement tool”) they can access without help. However, they felt that there are no substitutes for the provider-patient relationship, personalism in Latino culture, and loyalty to a provider: “...technology might not have the same impact.” Although intervention participants had the option to share their health promotion plan printout and discuss their study participation with their provider, the providers reported that none of the participants did. Therefore, a significant limitation on provider feedback about the CARE+ Spanish program was their lack of familiarity with the tool.

## Discussion

### Principal Results

The CARE+ computer-based counseling tool adapted for a Spanish-speaking population (CARE+ Spanish) was acceptable and feasible to implement in an urban clinic setting. Participants liked the ease of use and the sense of privacy and confidentiality that the computer-based counseling tool provided. The health care providers agreed that multiple approaches are needed and that the counseling tool can be an additional resource for HIV care and support. The CARE+ Spanish program demonstrated trends in positive impact in reducing viral loads, increasing ART adherence and decreasing risky sexual behaviors in three comprehensive care clinics in New York City. In the CARE+ Spanish trial, differences between arms were not statistically significant. In contrast, the CARE+ computer-based counseling tool, when evaluated in a university-affiliated public HIV clinic and a community-based AIDS service organization in Seattle, was found to be efficacious in reducing HIV-1 viral load and sexual transmission risk behaviors [6]. These two randomized clinical trials highlight the importance of targeting the right populations when adapting technology tools to support patient treatment engagement. Although other computer-based behavioral interventions delivered in a clinic setting have been found to improve self-reported antiretroviral adherence [26] and reduce risky sexual behaviors [27,28], to the best of our knowledge, CARE+ Spanish is the first non-English language computer-based counseling program to provide medication adherence support and promote positive prevention in a HIV-positive minority population. Another version of the counseling tool, CARE+ Kenya, linguistically and culturally adapted for use in clinic settings in Kenya (ClinicalTrials.gov: NCT01015989), may provide additional insights regarding the efficacy of computer-based counseling interventions across different populations.

### Limitations and Strengths

We draw from the qualitative data to provide potential explanation of the statistical lack of effect noted from this intervention in this population and setting. Exit interviews from intervention participants and focus groups with providers highlight the efficacy of computer-based counseling tools in overcoming adherence challenges experienced by culturally and linguistically diverse communities, especially stigma. A study that explored an intervention to engage PLHA to initiate ART, found that by sampling participants primarily from a clinic setting, they were encountering individuals who had already overcome many of the barriers to initiating and adhering to ARTs [29]. It is possible, although this was not explored, that our participants were also further along in the HIV treatment cascade [30], and this may have been a factor in reducing the effect of the CARE+ Spanish intervention. The computer-based audio-narrated risk assessment that both groups received at baseline may have been enough to support the maintenance of adherence in both groups, and the positive trend noted in the treatment group explained by the impact of the full intervention. Additionally, health provider acceptability is important for any ICT tool that aims to be incorporated into real-world practice. Therefore, although the CARE+ Spanish intervention was developed as a stand-alone computer-based counseling tool, participants did receive a printout of their session that they could use to initiate conversation with their provider about adherence support and risk reduction. Instead, providers were unfamiliar with the CARE+ Spanish program and reported that participants did not share the health plan printouts with them. Although not a specific outcome measure, the lack of familiarity with the specifics of the program and the session printout, from the providers, may highlight that the participants were not engaging their providers on this aspect of their self-care, and this may have been a factor in reducing the effect of the intervention. Potential improvement of this counseling intervention may be developing a mobile cloud-based platform to support users in self-motivated behavioral change for better health. Additionally, targeting the intervention to individuals with adherence and/or sexual transmission risk problems may be another way of improving the program’s effectiveness.

An innovative strength of our study is the use of peers to support ICT use in a clinic setting for an important and often-neglected population that is disproportionately affected by HIV disease burden. Peer involvement in programs designed to impact attitudes and behaviors have been shown to be effective [31,32].

### Conclusion

A computer-based counseling tool grounded in the TAM theoretical model and delivered in Spanish was acceptable and feasible to implement in a high-volume HIV clinic setting. It can provide evidence-based, linguistically appropriate ART adherence support without requiring additional staff time, bilingual status, or translation services. We found that language preferences and cultural acceptability of a computer-based counseling tool exist on a continuum in our urban Spanish-speaking population. Theoretical frameworks of technology’s usefulness in behavioral modification needs further exploration in other languages and cultures to determine where on the HIV care and treatment continuum these interventions may have the greatest impact.

## Appendix

Multimedia Appendix 1 Consort-Ehealth Checklist.

Multimedia Appendix 2 CARE+ Spanish Participant Interview.

Multimedia Appendix 3 CARE+ Spanish Topic Guide: Clinic Staff Focus Group.

## Abbreviations

AIDS: acquired immune deficiency syndrome
ART: antiretroviral therapy
CARE+: Computer Assessment & Rx Education for HIV-positives
HIV: human immunodeficiency virus
ICT: information and communication technology
PLHA: people living with HIV/AIDS
RCT: randomized controlled trial
TAM: Technology Acceptance Model
VAS: visual analog scale

## References

[1] Centers for Disease Control and Prevention (2013). Fact sheet: HIV among Hispanics/Latinos in the United States and dependent areas.

[2] Panel on Antiretroviral Guidelines for Adults and Adolescents (2016). Guidelines for the Use of Antiretroviral Agents in HIV-1-Infected Adults and Adolescents.

[3] World Health Organization (2003). Adherence to Long Term Therapies: Evidence for Action.

[4] Simoni JM, Pearson CR, Pantalone DW, Marks G, Crepaz N (2006). Efficacy of interventions in improving highly active antiretroviral therapy adherence and HIV-1 RNA viral load. A meta-analytic review of randomized controlled trials. J Acquir Immune Defic Syndr.

[5] Rueda S, Park-Wyllie LY, Bayoumi AM, Tynan AM, Antoniou TA, Rourke SB, Glazier RH (2006). Patient support and education for promoting adherence to highly active antiretroviral therapy for HIV/AIDS. Cochrane Database Syst Rev.

[6] Kurth AE, Spielberg F, Cleland CM, Lambdin B, Bangsberg DR, Frick PA, Severynen AO, Clausen M, Norman RG, Lockhart D, Simoni JM, Holmes KK (2014). Computerized counseling reduces HIV-1 viral load and sexual transmission risk: findings from a randomized controlled trial. J Acquir Immune Defic Syndr.

[7] Skeels MM, Kurth A, Clausen M, Severynen A, Garcia-Smith H (2006). CARE+ user study: usability and attitudes towards a tablet pc computer counseling tool for HIV+ men and women. AMIA Annu Symp Proc.

[8] Hayes-Bautista DE, Chapa J (1987). Latino terminology: conceptual bases for standardized terminology. Am J Public Health.

[9] (2013). Centers for Disease Control.

[10] Lai T, Larson EL, Rockoff ML, Bakken S (2008). User acceptance of HIV TIDES--Tailored Interventions for Management of Depressive Symptoms in persons living with HIV/AIDS. J Am Med Inform Assoc.

[11] Polgar S, Marshall J, Marshall J, Polgar S (1976). The search for culturally acceptable fertility regulating methods. Culture, Natality and Family Planning (Monograph 21).

[12] World Health Organization (1973). Task Force on Acceptability of Fertility Regulating Methods. Doc. ATF-G(2/73).

[13] Vincent D, McEwen MM, Pasvogel A (2008). The validity and reliability of a Spanish version of the summary of diabetes self-care activities questionnaire. Nurs Res.

[14] Jaja C, Pares-Avila J, Wolpin S, Berry D (2010). Usability evaluation of the interactive Personal Patient Profile-Prostate decision support system with African American men. J Natl Med Assoc.

[15] Fonteyn ME, Kuipers B, Grobe SJ (1993). A description of think aloud method and protocol analysis. Qual Health Res.

[16] Schulz KF, Altman DG, Moher D (2010). CONSORT 2010 statement: updated guidelines for reporting parallel group randomized trials. Ann Intern Med.

[17] Eysenbach G, CONSORT-EHEALTH Group (2011). CONSORT-EHEALTH: improving and standardizing evaluation reports of Web-based and mobile health interventions. J Med Internet Res.

[18] Fisher JD, Fisher WA, Amico KR, Harman JJ (2006). An information-motivation-behavioral skills model of adherence to antiretroviral therapy. Health Psychol.

[19] Bandura A (2004). Health promotion by social cognitive means. Health Educ Behav.

[20] Tober G (2013). Motivational interviewing: helping people change. Alcohol Alcoholism.

[21] Thibault JM, Steiner RW (2004). Efficient identification of adults with depression and dementia. Am Fam Physician.

[22] Faulkner L (2003). Beyond the five-user assumption: benefits of increased sample sizes in usability testing. Behav Res Methods Instrum Comput.

[23] Bates D, Mächler M, Bolker B, Walker S (2015). Fitting linear mixed-effects models using lme4. J Stat Soft.

[24] R Development Core Team R-A language and environment for statistical computing.

[25] Selya AS, Rose JS, Dierker LC, Hedeker D, Mermelstein RJ (2012). A practical guide to calculating Cohen's f(2), a measure of local effect size, from PROC MIXED. Front Psychol.

[26] Fisher JD, Amico KR, Fisher WA, Cornman DH, Shuper PA, Trayling C, Redding C, Barta W, Lemieux AF, Altice FL, Dieckhaus K, Friedland G, LifeWindows Team (2011). Computer-based intervention in HIV clinical care setting improves antiretroviral adherence: the LifeWindows Project. AIDS Behav.

[27] Grimley DM, Hook EW (2009). A 15-minute interactive, computerized condom use intervention with biological endpoints. Sex Transm Dis.

[28] Lightfoot M, Rotheram-Borus MJ, Comulada WS, Reddy VS, Duan N (2010). Efficacy of brief interventions in clinical care settings for persons living with HIV. J Acquir Immune Defic Syndr.

[29] Gwadz M, Cleland CM, Applegate E, Belkin M, Gandhi M, Salomon N, Banfield A, Leonard N, Riedel M, Wolfe H, Pickens I, Bolger K, Bowens D, Perlman D, Mildvan D, Heart to Heart Collaborative Research Team (2015). Behavioral intervention improves treatment outcomes among HIV-infected individuals who have delayed, declined, or discontinued antiretroviral therapy: a randomized controlled trial of a novel intervention. AIDS Behav.

[30] Gardner EM, McLees MP, Steiner JF, Del RC, Burman WJ (2011). The spectrum of engagement in HIV care and its relevance to test-and-treat strategies for prevention of HIV infection. Clin Infect Dis.

[31] Chapman DJ, Damio G, Young S, Pérez-Escamilla R (2004). Effectiveness of breastfeeding peer counseling in a low-income, predominantly Latina population: a randomized controlled trial. Arch Pediatr Adolesc Med.

[32] Kelly JA, St Lawrence JS, Diaz YE, Stevenson LY, Hauth AC, Brasfield TL, Kalichman SC, Smith JE, Andrew ME (1991). HIV risk behavior reduction following intervention with key opinion leaders of population: an experimental analysis. Am J Public Health.

