# Supplementary material for: Linguistic and Cultural Adaptation of a Computer-Based Counseling Program (CARE+ Spanish) to Support HIV Treatment Adherence and Risk Reduction for People Living With HIV/AIDS: A Randomized Controlled Trial
Source: J Med Internet Res. 2016 Jul 13;18(7):e195. doi: 10.2196/jmir.5830 (PMC4963608; doi:10.2196/jmir.5830)
Supplement: Multimedia Appendix 2 [file jmir_v18i7e195_app2.pdf]

CARE+ Spanish Participant Interview

Date of Interview: ___/__/__ Location: ________________ Interviewer (initials): ___ Client Study ID:_________

- Review study purpose, consent form, answer questions. Explain no audiotaping or name kept with notes – only study ID.

Client Age:______ Country of Birth:________________Number of years living in the US_______

Number of years in HIV/AIDS care:___________

1. **Currently what are the most urgent issues you face in your HIV/AIDS care?** (Note: most pressing, most urgent issues in general)

2. **Do you think these problems/concerns are different for Latino/as compared with non-Latino/as living with HIV/AIDS? Why/why not?**

3. **Have you told anyone in your life about your HIV status?** (*Probe:* who, why/why not)

4. **What do you think is the best way for people like yourself to get health information?** *(Probe: talking with family, peers, health workers, religious leaders, radio, television, brochures, computer/cell phone, other)*

5. **Of the sources we just talked about:**
   a) **Which do you trust the most to get information about HIV/AIDS?**

   b) **Why is that a good source?**

6. **What about information on ways to stay healthy living with HIV/AIDS?** (Note: Refer back to the sources mentioned in question 5; Probe if different source of information)

7. **What have your clinic providers (all providers that they encounter as part of their clinic visit- can be the social worker, peers etc.) told you about**:
   a) **Why it is important to take your HIV/AIDS meds correctly?**

   b) **Having a sexual life with HIV/AIDS?**

   c) **How to lower the chance of passing HIV on to sex partners or babies?**

8. **Do you feel comfortable asking questions about:**
   a) **medications?**

   b) **issues about sexual behavior?** (Probe why if no; if yes, probe what makes them feel comfortable asking questions, e.g. provider characteristics)

9. In this project you used a computer to get information about living with HIV/AIDS.
a) What did you think about "talking" with the computer here at the clinic?

b) What did you like most about using the computer?

c) What did you like least about using the computer?

d) What language would you prefer for listening to the computer voice?
(English or Spanish)

10. How anxious were you about using the computer <u>at the study **START**?</u> *(Probe open-ended first, then rating)*

**Anxious** Level	**0** Not at all anxious	**1** Somewhat anxious	**2** Very anxious

11. How anxious were you about using the computer <u>at the study **END**?</u> *(Probe open-ended first, then rating)*

**Anxious?** Level	**0** Not at all anxious	**1** Somewhat anxious	**2** Very anxious

12. (If these ratings differ, ask): **What changed your thinking about this?**

13. **What did you think about the confidentiality of having the computer talk to you through headphones?**

14. **What did you think about having Spanish narration?**

15. **What did you think about having peers (other Latino/as living with HIV/AIDS) explain the study?**

16. **What did you think about having peers (other Latino/as living with HIV/AIDS) explain how to use the computer?**

17. Now that the study is finished, do you think you would use the CARE+ Spanish tool on your own if the computers were here in the clinic?

a) If yes, how often do you think you would use it?

i. Why would you use it?

ii. What would help make it a regular part of your clinic visit?

b) If no, why not?

- **Please suggest any improvements** for using computer counseling in the clinic.

Is there anything else that you would like us to know or that you think is important to make this a better tool or easier to use?

Thank you for sharing your valuable time and helping us with this study.
