# Supplementary material for: Linguistic and Cultural Adaptation of a Computer-Based Counseling Program (CARE+ Spanish) to Support HIV Treatment Adherence and Risk Reduction for People Living With HIV/AIDS: A Randomized Controlled Trial
Source: J Med Internet Res. 2016 Jul 13;18(7):e195. doi: 10.2196/jmir.5830 (PMC4963608; doi:10.2196/jmir.5830)
Supplement: Multimedia Appendix 3 [file jmir_v18i7e195_app3.pdf]

CARE+ Spanish Topic Guide: Clinic Staff Focus Group

Date of Interview: ___/__/__ Location: _________________ 1st facilitator (initials): ___ 2nd facilitator:____

- Interviewer: make introductions, explain what to expect, ground rules for confidentiality, and that we are taking notes, not audiotaping.
- Review study purpose, consent form; answer questions, obtain signatures.

Draw map /seating chart, noting location, sex, provider type

1. **Current issues in provision of care to HIV+ patients**
   - Currently what are the most urgent issues you face in providing care to HIV+ patients? Is it any different for Latino/as?

     How do these issues affect you as care providers?

     How do these issues affect HIV+ patients?

   [Probe strengths of program/facility and challenges and how they affect Latino population specifically]

2. **Counseling messages regarding 'prevention with positives' and adherence**
   - What challenges do health providers face in dealing with ART adherence issues? Are these challenges different for Latino patients? Different groups of Latino patients?

   - What institutional supports does your program have in addressing adherence obstacles for patients?

   - What are some of the counseling messages regarding 'prevention with positives' that is, how do you or your colleagues address the sexual risk (of getting STIs or HIV re-infection) with your HIV+ patients?

     What is the message given to HIV-positive patients about using condoms or other HIV transmission risk reduction practices? Materials provided?

   - What challenges do health providers face in providing positive prevention? Is it any different for Latino/a patients?

     What are the most difficult situations faced when discussing prevention with positives?
     What about with Latino/as specifically?

3. **Computer tools for delivering counseling**
   - How do you think our using the CARE+ Spanish computer tool to deliver counseling to patients went?

   - Were there any problems/barriers in doing computer counseling here? What/Why? How was it addressed?

   - What were the likely benefits to doing computer counseling here? Did you identify anything specifically?

4. What is your perception of the usefulness of this tool in the clinic? (solicit open-ended responses first, then ask each person to choose Likert scale rating: write scale number on slip of paper, fold it & hand it in)

Usefulness? Level	0	1	2	3	4
	Not at all useful	Somewhat not useful	Average usefulness	Somewhat useful	Very useful

5. **What is your perception of the <u>ease of use</u>** of this tool in the clinic? *<u>By</u>* **<u>patients</u>**: (solicit open-ended responses first, then ask each person to choose Likert scale rating, write scale number on slip of paper, fold it & hand it in)

**Ease of use BY PTS?** Level	**0** Not at all easy	**1** Somewhat not easy	**2** Average ease	**3** Somewhat easy	**4** Very easy

<u>In terms of impact on **providers**</u>, overall?

**Impact on Providers?** Level	**0** Not at all problematic	**1** Somewhat problematic	**2** Average	**3** Somewhat helpful	**4** Very helpful

<u>In terms of impact on **clinic operations and patient flow**</u>, overall?

**Impact on CLINIC?** Level	**0** Not at all problematic	**1** Somewhat problematic	**2** Average	**3** Somewhat helpful	**4** Very helpful

6. **How do you think this CARE+ Spanish computer tool may have impacted provider-patient communication?**

7. **How do you think this CARE+ Spanish computer tool may have impacted** staff demand and resource use?

8. **Now that the study is done, would you want to see the clinic continue to use the CARE+ Spanish tool** (i.e., keep the computers here in the clinic for patients to use on their own)?

   If yes, how often do you think patient would use it?

   What would help make it a regular part of a clinic visit?

   If no, why not?

- **Please suggest any improvements** for using computer counseling in the clinic.

Is there anything else that you would like us to know or that you think is important?

Thank you for your interest and comments. We appreciate your valuable time!
